# Investigation of the Genus *Flavobacterium* as a Reservoir for Fish-Pathogenic Bacterial Species: the Case of Flavobacterium collinsii

**DOI:** 10.1128/aem.02162-22

**Published:** 2023-03-28

**Authors:** Bo-Hyung Lee, Pierre Nicolas, Izzet Burcin Saticioglu, Benjamin Fradet, Jean-François Bernardet, Dimitri Rigaudeau, Tatiana Rochat, Eric Duchaud

**Affiliations:** a Université Paris-Saclay, INRAE, UVSQ, VIM, Jouy-en-Josas, France; b Université Paris-Saclay, INRAE, MaIAGE, Jouy-en-Josas, France; c Department of Aquatic Animal Diseases, Faculty of Veterinary Medicine, Bursa Uludag University, Bursa, Turkey; d Université Paris-Saclay, IERP, INRAE, Jouy-en-Josas, France; INRS Armand-Frappier Sante Biotechnologie Research Centre

**Keywords:** *Flavobacterium*, *Flavobacterium collinsii*, aquaculture, experimental challenges, fish pathogens, opportunistic pathogens, rainbow trout, virulence

## Abstract

Bacteria of the genus *Flavobacterium* are recovered from a large variety of environments. Among the described species, Flavobacterium psychrophilum and Flavobacterium columnare cause considerable losses in fish farms. Alongside these well-known fish-pathogenic species, isolates belonging to the same genus recovered from diseased or apparently healthy wild, feral, and farmed fish have been suspected to be pathogenic. Here, we report the identification and genomic characterization of a Flavobacterium collinsii isolate (TRV642) retrieved from rainbow trout spleen. A phylogenetic tree of the genus built by aligning the core genome of 195 *Flavobacterium* species revealed that *F. collinsii* stands within a cluster of species associated with diseased fish, the closest one being F. tructae, which was recently confirmed as pathogenic. We evaluated the pathogenicity of *F. collinsii* TRV642 as well as of Flavobacterium bernardetii F-372^T^, another recently described species reported as a possible emerging pathogen. Following intramuscular injection challenges in rainbow trout, no clinical signs or mortalities were observed with *F. bernardetii*. *F. collinsii* showed very low virulence but was isolated from the internal organs of survivors, indicating that the bacterium is able to survive inside the host and may provoke disease in fish under compromised conditions such as stress and/or wounds. Our results suggest that members of a phylogenetic cluster of fish-associated *Flavobacterium* species may be opportunistic fish pathogens causing disease under specific circumstances.

**IMPORTANCE** Aquaculture has expanded significantly worldwide in the last decades and accounts for half of human fish consumption. However, infectious fish diseases are a major bottleneck for its sustainable development, and an increasing number of bacterial species from diseased fish raise a great concern. The current study revealed phylogenetic associations with ecological niches among the *Flavobacterium* species. We also focused on Flavobacterium collinsii, which belongs to a group of putative pathogenic species. The genome contents revealed a versatile metabolic repertoire suggesting the use of diverse nutrient sources, a characteristic of saprophytic or commensal bacteria. In a rainbow trout experimental challenge, the bacterium survived inside the host, likely escaping clearance by the immune system but without provoking massive mortality, suggesting opportunistic pathogenic behavior. This study highlights the importance of experimentally evaluating the pathogenicity of the numerous bacterial species retrieved from diseased fish.

## INTRODUCTION

Members of the genus *Flavobacterium* (phylum *Bacteroidota*, family *Flavobacteriaceae*) are most frequently isolated from environmental sources. They are common in freshwater environments and in soil, especially in the rhizosphere, but strains have also been isolated from brackish water or seawater and glaciers in temperate, tropical, and polar areas (1). Following new molecular omics-based approaches, the taxonomy has been clarified and the number of formally described *Flavobacterium* species has rapidly expanded to include 268 species at the time of writing (https://lpsn.dsmz.de/genus/Flavobacterium [accessed August 2022]).

The vast majority of these species are considered harmless, consuming inert organic matter and thus playing an important role in biogeochemical cycles (2). Among them, Flavobacterium johnsoniae has emerged as a model organism for studying gliding motility and protein secretion (3). In addition, the genus encompasses two very important fish pathogens, F. psychrophilum and F. columnare, both reported to cause considerable losses in farmed and wild freshwater fish. To account for its genomic and phenotypic diversity, the latter has been recently divided into four distinct species, namely, F. columnare, F. covae, F. davisii, and F. oreochromis, based on genomic comparisons as well as differences in host fish species and virulence (4). Another species, F. branchiophilum, is also known as a fish pathogen, but within more restricted geographical areas. In addition, the following species, often represented by a very small number of isolates, were recovered from diseased fish tissues and have been suspected to be pathogenic: F. araucananum from kidney and external lesions of Atlantic salmon (Salmo salar) (5); F. bernardetii from kidney and liver of rainbow trout (Oncorhynchus mykiss) (6); F. turcicum and F. kayseriense from rainbow trout kidney and spleen, respectively (7); F. branchiarum and F. branchiicola from rainbow trout gills (8); F. chilense from external lesions of rainbow trout (5); F. collinsii from the liver of rainbow trout (8); F. hydatis from the gills of diseased salmon (9, 10); F. inkyongense from diseased chocolate cichlids (Hypselecara coryphaenoides) (11); F. johnsoniae-like isolates from various diseased fish species (12); F. oncorhynchi from liver and gills of rainbow trout (13); F. piscis from liver, gills, and kidney of rainbow trout (14); F. plurextorum from liver and eggs of rainbow trout (15); and F. succinicans from gills of rainbow trout suffering bacterial gill disease (16). At least one species, F. tructae, which was isolated from liver, gills, and kidney of rainbow trout (14) and concurrently from kidney of feral spawning adult Chinook salmon (Oncorhynchus tshawytscha) under the alternative name of F. spartansii (17), may be considered a salmonid pathogen, as two isolates were able to induce pathological changes and mortality in experimentally infected Chinook salmon, though only using very high infectious doses (18). In contrast, most of the aforementioned fish-associated species have not been assessed for their level of virulence using experimental challenges.

The lack of information about the pathogenicity of many *Flavobacterium* species can lead to unnecessary and irrational use of antimicrobials and, conversely, to the lack of surveillance of bacteria that can cause disease outbreaks. Interestingly, the continuous increase in whole-genome sequencing generates data that may help identifying ecological niches and pathogenicity, by providing improved phylogenetic resolution and access to gene repertoires underpinning the phenotypes. As an initiative for this approach in the genus *Flavobacterium*, we exploited the large number of genomes available to investigate the relationship between phylogeny and environmental niches at the species level. In parallel and in attempt to satisfy Koch’s postulates, we assessed the virulence of two recent isolates of *F. bernardetii* (6) and *F. collinsii*, a species closely related to *F. tructae*, using experimental infection in rainbow trout.

## RESULTS

### A new *F. collinsii* isolate retrieved from rainbow trout.

In order to survey the health status of rainbow trout raised at INRAE fish facilities (IERP), different organs of dead fish are regularly sampled to perform basic bacteriological quality controls. Organs (i.e., usually spleen and liver) are crushed in tryptone-yeast extract-salt (TYES) broth and the lysates are spread on petri dishes to ensure the absence of any pathogenic *Flavobacterium* species. Surprisingly, during the summer of 2020, yellow-pigmented bacterial colonies with high spreading activity were observed after streaking the spleen of one rainbow trout fingerling (2 g) that had died without any disease symptom. The fish belonged to the highly F. psychrophilum-susceptible A36 isogenic line (19). A pure culture was obtained from one bacterial colony and 16S rRNA gene sequencing identified the bacterium as probably belonging to the species *F. collinsii*. For a more accurate characterization, the complete genome of this isolate, named TRV642, was resolved by high-throughput sequencing using the hybrid assembly of long Nanopore reads and short Illumina reads. The average nucleotide identity (ANI) between strain TRV642 and the *F. collinsii* type strain, CECT 7796, was 98.30%, far above the threshold delineation (cutoff, 95 to 96%) of a bacterial species (20), thus confirming the initial 16S rRNA gene taxonomic affiliation.

### Phylogenetic analysis and correlation with the ecological niche at the genus level.

**FIG 1:**
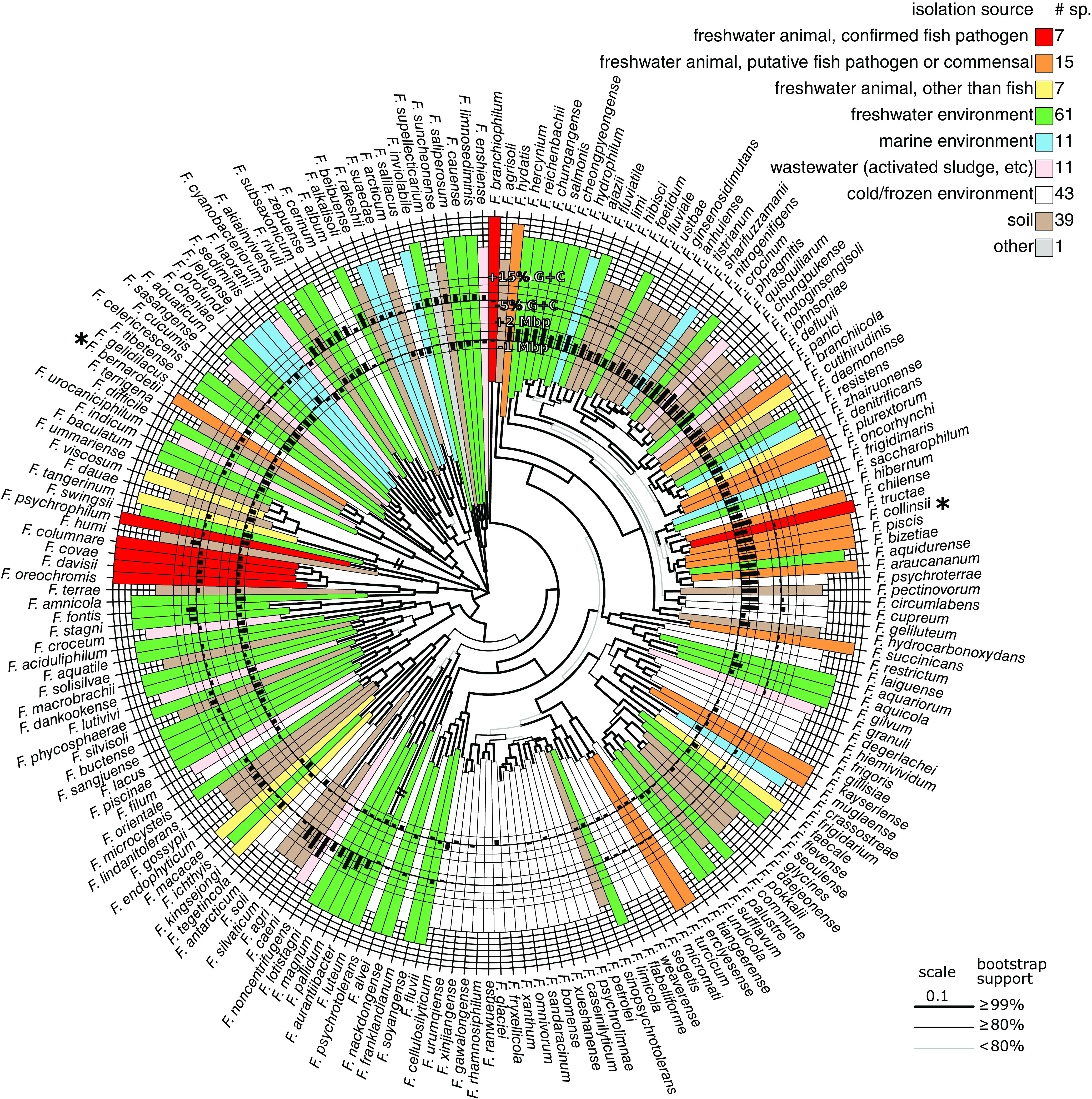
Tentative phylogenetic tree of 195 *Flavobacterium* species. The tree is based on the protein sequences of the 510 single-copy genes of all *Flavobacterium* species for which the complete genome is available in public databases. For clarity, the tree was midpoint rooted. Genome size and G+C contents are compared to the medians for the genus and shown as circular bar plots. Color and position on outer circle indicate the source of isolation of the type strain, with confirmed pathogen species in red and outermost position.

To cover the diversity of the species with complete genome records available in public databases, we extracted from the RefSeq database all genomes belonging to genus *Flavobacterium* with the tag “representative,” constituting a set of 195 genomes from distinct species. Gene repertoire comparisons delineated a core of 510 conserved single-copy genes. Their multiple-sequence alignment after gap removal represented 154,805 amino acids (98,363 polymorphic sites) and was used for phylogenetic reconstruction. A tentative phylogenetic tree for the 195 *Flavobacterium* species is shown in Fig. 1. The genome size and G+C content in comparison to the corresponding medians for the genus are also shown in Fig. 1.

To examine the links between position in the phylogenetic tree and ecological niche, we retrieved the environment from which the type strain was sampled from the original publication describing each species. The diversity of environments was summarized into 9 different categories (Fig. 1; see also Data Set S1 in the supplemental material), distinguishing 3 categories for the 29 species isolated from animal-associated samples, 5 categories for the 165 species isolated from other important natural environments, and 1 category, “other,” to account for the single species isolated in a totally different and more artificial context (F. supellecticarium isolated from a “synthetic wooden board” (21)).

According to this classification, the 165 species (84.6% of the total) originating from non-animal-related environmental samples were composed of 60 species (30.8%) retrieved from freshwater, 45 species (23.1%) from cold/frozen environments (predominantly Antarctic soil or glacier), 38 species (19.5%) from soils, 11 species (5.6%) species from marine environments (including sea sediments and algae), and 11 species (5.6%) from wastewater-related environments. Out of the 29 species (14.9% of the total) retrieved from, or in contact with, animals, only 7 of these animals were not fish. The 22 fish-associated species (11.3% of the total) were further divided into 7 confirmed pathogens (4 of which were previously considered genomovars of *F. columnare*) and 15 putative pathogens or fish commensals. Taken together, the classification of the environments and the phylogenetic tree shed light on the possible connections between evolutionary links and ecological niches in the genus *Flavobacterium*. Among the patterns that emerge from this picture, a group around F. glaciei is mostly composed of species retrieved from cold/frozen environments. Some links between phylogenetic position and genome characteristics were also observed. In terms of G+C content, 8 species with particularly high G+C contents form a phylogenetic group around F. caeni. In terms of genome size, a large phylogenetic group encompassing more than a quarter of all considered species (51 species [26.2%]), including *F. johnsoniae*, displays genomes up to 2 Mbp larger than the median value for the genus.

Whatever the specific category considered (“fish pathogen,” “fish associated,” or “animal other than fish”), species associated with animals are scattered across the phylogenetic tree. Exceptions are the 4 fish-pathogenic species resulting from the splitting of *F. columnare* (which group together), 2 pairs of closely related “fish-related species” (*F. plurextorum*-*F. oncorhynchi* and F. erciyesense-*F. turcicum*), and a more important cluster of species around *F. tructae* that includes *F. collinsii*.

### *F. collinsii* belongs to a cluster of fish-associated species with large genomes.

Strikingly, *F. collinsii* is the closest relative of the confirmed pathogen *F. tructae* but the two species are well distinct, as confirmed by the ANI of only 87.56% between *F. collinsii* TRV642 and *F. tructae* MSU. These two species belong to a cluster of putative pathogenic species with *F. araucananum*, F. bizetiae, *F. piscis*, and *F. chilense*. All these species were isolated from diseased salmonid fish except for F. bizetiae, whose origin of isolation was more loosely described as “a diseased freshwater fish” (22). All these species are predicted to derive from a common ancestor, also shared with F. aquidurense, F. psychroterrae, and F. hibernum, 3 species not retrieved from animal-related material. This cluster, enriched in fish-related species comprising *F. collinsii* and *F. tructae*, belongs to the above-mentioned large genome group comprising *F. johnsoniae*.

The complete genome of *F. collinsii* TRV642 consists of a circular chromosome of 5,554,530 bp and predicted to contain 4,285 coding DNA sequences (CDS), 69 tRNA genes, and 7 rRNA operons. The genome size is slightly smaller (by 181,002 bp) than that of the *F. collinsii* type strain, CECT 7796. All species belonging to the aforementioned cluster comprising *F. tructae* also possess large genomes (from 5.4 to 6.1 Mbp), comparable to the 6.1-Mbp genome of the well-studied environmental species *F. johnsoniae* (23). These genomes are about 2-fold larger than the 2.9-Mbp genome of F. psychrophilum (24) and the 3.2-Mbp genome of the *F. columnare* type strain, ATCC 23463 (4); these two species are well characterized and unquestionably fish pathogenic.

### Insights from the gene repertoire.

**(i) Protein secretion.** The type IX secretion system (T9SS) is responsible for protein secretion and required for gliding motility (3). This machinery is confined to the phylum *Bacteroidota*, and genes encoding the core components of the T9SS machinery, the attachment complex, and the *gld* genes needed for gliding motility were all identified in the *F. collinsii* TRV642 genome (Data Set S2).

Proteins secreted by the T9SS possess conserved C-terminal domains (CTDs). In the *F. collinsii* TRV642 genome, 45 genes encoding proteins with a type A (TIGR04183) CTD were identified (Data Set S3), some with predicted enzymatic functions, as well as 9 genes encoding proteins with a type B (TIGR04131) CTD, none with predicted enzymatic function. Enzymes with a type A CTD include 9 peptidases likely involved in protein/peptide degradation, all conserved in the genomes of *F. tructae* and *F. chilense* but absent, with a few exceptions, in those of F. psychrophilum and *F. columnare*. Among the other type A CTD enzymes, two are polysaccharide lyases and one is a glycoside hydrolase likely involved in carbohydrate degradation. This repertoire of T9SS-secreted enzymes supports the view of *F. collinsii* as a degrader of high-molecular-weight organic matter and suggests metabolic versatility, a functional trait shared by some members of the family *Flavobacteriaceae*, such as *F. johnsoniae* (23).

In addition to the *Bacteroidota*-specific T9SS, a B-type T4SS is also present in the *F. collinsii* TRV642 genome (Data Set S2). This versatile secretion system is utilized to mediate horizontal gene transfer and also allows Gram-negative pathogenic bacteria to translocate a wide variety of virulence factors into the host cell (25). Because the *F. collinsii* T4SS locus encompasses genes involved in DNA transfer and encoding relaxase/mobilization family proteins (i.e., MobA/VirD2 and MobC), it is tempting to speculate that the *F. collinsii* T4SS is dedicated to the recruitment and delivery of DNA substrates.

**(ii) PUL and carbohydrate-active enzymes (CAZymes).** Members of the phylum *Bacteroidota* have developed multicomponent protein systems aimed at sensing, binding, transporting, and degrading specific glycans (26). Genes encoding these systems are often colocalized in regions referred to as polysaccharide utilization loci (PUL). Tandem *susD*-like and *susC*-like genes, which encode a carbohydrate-binding lipoprotein and a TonB-dependent transporter, respectively, are considered a hallmark of PUL, and these *susCD*-like gene pairs are generally used to identify PUL in *Bacteroidota* genomes (27). It has been suggested that *susCD*-like pairs could transport substrates other than carbohydrates (23), and this was recently confirmed by the finding that the BT2263-2264 pair of Bacteroides thetaiotaomicron and the RagAB transporter of Porphyromonas gingivalis import oligopeptides (28, 29).

The *F. collinsii* TRV642 genome contains 29 *susCD*-like pairs, most of which are encompassed in bona fide PUL, namely, with adjacent genes encoding obvious additional polysaccharide utilization proteins (i.e., CAZymes) (Table 1; see also the figure in the supplemental material). About half of these PUL have been previously identified in the *F. johnsoniae* UW101 genome (23). For instance, PUL TRV642_1394 to -1405 are predicted to be dedicated to α-glucan/starch degradation (corresponding to PUL Fjoh_1398 to -1408), PUL TRV642_1594 to -1602 to β-glucan/xylan degradation (corresponding to PUL Fjoh_1559 to -1567), and PUL TRV642_4202 to -4209 to chitin degradation (corresponding to PUL Fjoh_4555 to -4564). In addition to these PUL shared with *F. johnsoniae*, some PUL are likely of functional importance. For instance, PUL TRV642_0198 to -0209 encompass 5 peptidases (belonging to families S9, S37, M24, and M43) and are likely involved in the uptake of oligopeptides. PUL TRV642_4544 to -4556 encompass 12 genes, among which are TRV642_4547, encoding a polysaccharide lyase (family PL8) protein precursor containing a type A T9SS C-terminal secretion signal likely anchored in the outer membrane, facing the extracellular milieu, and *hepC*, encoding a protein highly similar (63% amino acid identity) to heparinase III of the nonpathogenic soil bacterium Pedobacter heparinus, degrading heparan sulfate (30) and likely located in the periplasm. These two genes, organized in tandem, as well as other glycoside hydrolase (GH)-, polysaccharide lyase (PL)- and sulfatase-encoding genes, are likely involved in the degradation of complex sulfated carbohydrates belonging to the glycosaminoglycan family, such as heparan sulfate.

**TABLE 1:** *susCD* pairs identified in the Flavobacterium collinsii TRV642 genome

Locus tag	Product	InterPro identifier(s)	In a *bona fide* PUL	Predicted substrate
TRV642_0169	SusC-like protein	IPR023996 and IPR023997	No	Unknown
TRV642_0168	SusD-like protein	IPR012944 and IPR033985		
TRV642_0202	SusC-like protein	IPR023996 and IPR023997	Yes	Oligopeptide
TRV642_0201	SusD-like protein	IPR041662		
TRV642_0403	SusC-like protein	IPR023996 and IPR023997	No	Unknown
TRV642_0404	SusD-like protein	IPR041662		
TRV642_0405	SusC-like protein	IPR023996 and IPR023997	No	Unknown
TRV642_0406	SusD-like protein	IPR041662		
TRV642_0407	SusC-like protein	IPR023996 and IPR023997	No	Unknown
TRV642_0408	SusD-like protein	IPR041662		
TRV642_0663	SusC-like protein	IPR023996 and IPR023997	No	Unknown
TRV642_0664	SusD-like protein	IPR041662		
TRV642_0665	SusC-like protein	IPR023996 and IPR023997	No	Unknown
TRV642_0666	SusD-like protein	IPR041662		
TRV642_1402	SusC-like protein	IPR023996 and IPR023997	Yes	α-Glucans/starch
TRV642_1403	SusD-like protein	IPR012944 and IPR033985		
TRV642_1595	SusC-like protein	IPR023996 and IPR023997	Yes	β-Glucans/xylan
TRV642_1596	SusD-like protein	IPR012944 and IPR033985		
TRV642_1932	SusC-like protein	IPR023996 and IPR023997	Yes	Oligosaccharides
TRV642_1933	SusD-like protein	IPR041662		
TRV642_1968	SusC-like protein	IPR023996 and IPR023997	No	Unknown
TRV642_1969	SusD-like protein	IPR012944 and IPR033985		
TRV642_2080	SusC-like protein	IPR023996 and IPR023997	No	Unknown
TRV642_2081	SusD-like protein	IPR012944 and IPR033985		
TRV642_2255	SusC-like protein	IPR023996 and IPR023997	No	Unknown
TRV642_2254	SusD-like protein	IPR033985		
TRV642_2325	SusC-like protein	IPR023996 and IPR023997	No	Unknown
TRV642_2326	SusD-like protein	IPR041662		
TRV642_2567	SusC-like protein	IPR023996 and IPR023997	Yes	Oligosaccharides
TRV642_2566	SusD-like protein	IPR012944 and IPR033985		
TRV642_2576	SusC-like protein	IPR023996 and IPR023997	No	Unknown
TRV642_2575	SusD-like protein	IPR012944 and IPR033985		
TRV642_2869	SusC-like protein	IPR023996 and IPR023997	Yes	Oligosaccharides
TRV642_2870	SusD-like protein	RagB/SusD domain		
TRV642_3296	SusC-like protein	IPR023996 and IPR023997	No	Unknown
TRV642_3295	SusD-like protein	IPR033985		
TRV642_3433	SusC-like protein	IPR023996 and IPR023997	No	Unknown
TRV642_3432	SusD-like protein	IPR041662		
TRV642_3468	SusC-like protein	IPR023996 and IPR023997	Yes	α-Glucans
TRV642_3467	SusD-like protein	IPR012944 and IPR033985		
TRV642_3614	SusC-like protein	IPR023996 and IPR023997	No	Unknown
TRV642_3613	SusD-like protein	IPR012944 and IPR033985		
TRV642_3711	SusC-like protein	IPR023996 and IPR023997	Yes	Oligosaccharides
TRV642_3712	SusD-like protein	IPR012944 and IPR033985		
TRV642_3733	SusC-like protein	IPR023996 and IPR023997	Yes	Pectins
TRV642_3732	SusD-like protein	IPR012944 and IPR033985		
TRV642_3861	SusC-like protein	IPR023996 and IPR023997	Yes	Oligopeptide
TRV642_3860	SusD-like protein	IPR012944 and IPR033985		
TRV642_4207	SusC-like protein	IPR023996	Yes	Chitin
TRV642_4206	SusD-like protein	IPR024302		
TRV642_4321	SusC-like protein	IPR023996 and IPR023997	No	Unknown
TRV642_4322	SusD-like protein	IPR012944 and IPR033985		
TRV642_4333	SusC-like protein	IPR023996 and IPR023997	No	Unknown
TRV642_4334	SusD-like protein	IPR012944 and IPR033985		
TRV642_4524	SusC-like protein	IPR023996 and IPR023997	Yes	Oligopeptide
TRV642_4525	SusD-like protein	IPR012944 and IPR033985		
TRV642_4549	SusC-like protein	IPR023996 and IPR023997	Yes	Heparin/chondroitin sulfate
TRV642_4550	SusD-like protein	IPR012944 and IPR033985		

Genome analysis revealed a large gene repertoire (122) of carbohydrate-active enzyme (CAZymes) modules encompassed in 112 genes. Strikingly, this gene repertoire is approximately two times larger than in representative strains of pathogenic species F. psychrophilum (56) and *F. columnare* (63) (Table 2). Overrepresentation is even more obvious when CAZymes dedicated solely to carbohydrate degradation are taken into account; for example, the *F. collinsii* genome encodes 51 GHs and 9 PLs. In contrast, the F. psychrophilum JIP02/86 genome contains 8 GHs and 0 PLs and the *F. columnare* ATCC 49512 genome contains 13 PLs and 3 PLs. The high number of genes encoding CAZymes is a trait shared with the genomes of *F. tructae* and the cluster of putative pathogenic species (i.e., *F. collinsii*, *F. araucananum*, *F. bizetiae*, *F. piscis*, and *F. chilense*). These numbers range from 112 for *F. collinsii* to 238 for *F. piscis* (Table 2), the latter being comparable to the number found in the environmental bacterium *F. johnsoniae* (243). Therefore, data from T9SS-secreted proteins, PUL systems, and CAZymes indicate that *F. collinsii* evolved to take advantage of a large variety of both proteinaceous and carbohydrate substrates.

**TABLE 2:** Number of CAZyme modules encoded by *Flavobacterium* species

Species	Strain	Environment(s)	Genome size (Mbp)	No. of:						
				Glycoside hydrolases	Glycosyl transferases	Polysaccharide lyases modules	Carbohydrate esterases	Carbohydrate-binding modules	Total modules	Total genes
Flavobacterium johnsoniae	UW101	Freshwater, soil	6.09	157	57	12	13	20	259	243
Flavobacterium psychrophilum	JIP02/86	Pathogenic essentially for *Salmonidae*	2.86	8	44	0	4	2	58	56
Flavobacterium branchiophilum	FL-15	Pathogenic essentially for *Salmonidae*	3.55	26	52	2	5	4	89	85
Flavobacterium columnare	ATCC 49512	Pathogenic for many freshwater fish species	3.16	13	42	3	4	3	65	63
Flavobacterium collinsii	TRV642	Putative pathogen	5.55	51	48	9	7	7	122	112
Flavobacterium chilense	DSM 24724	Putative pathogen	6.11	124	45	14	11	22	216	198
*Flavobacterium tructae*	MSU	Putative pathogen	5.35	50	50	7	7	7	121	113
Flavobacterium piscis	CCUG 60099	Putative pathogen	5.9	162	50	10	12	17	251	238
*Flavobacterium bizetiae*	CIP 105534	Putative pathogen	5.87	149	50	23	13	17	252	222
Flavobacterium araucananum	DSM 24704	Putative pathogen	6.01	108	48	7	8	20	191	174

**(iii) Antibiotic biosynthesis.** The *F. collinsii* genome contains two large gene clusters (203 kb for TRV642_2161 to -2233 and 54 kb for TRV642_4252 to -4268) encoding nonribosomal peptide/polyketide synthase enzymes likely involved in antibiotic biosynthesis. The genomes of *F. tructae* and *F. johnsoniae* also contain antibiotic biosynthesis gene clusters, whereas those of F. psychrophilum and *F. columnare* are devoid of these loci, as well as of other antibiotic biosynthesis-encoding genes.

### Virulence assessment in rainbow trout.

With the increasing number of *Flavobacterium* species isolated from diseased fish worldwide (31), a better characterization of their lifestyle and their impact on fish health is needed. Within the time frame of this study, a novel *Flavobacterium* species named *F. bernardetii* was described after the recovery of two isolates from diseased rainbow trout exhibiting neurological symptoms (6). Together with *F. collinsii*, this species was proposed as a possible pathogen, although pathogenicity was not properly validated using experimental infection.

**FIG 2:**
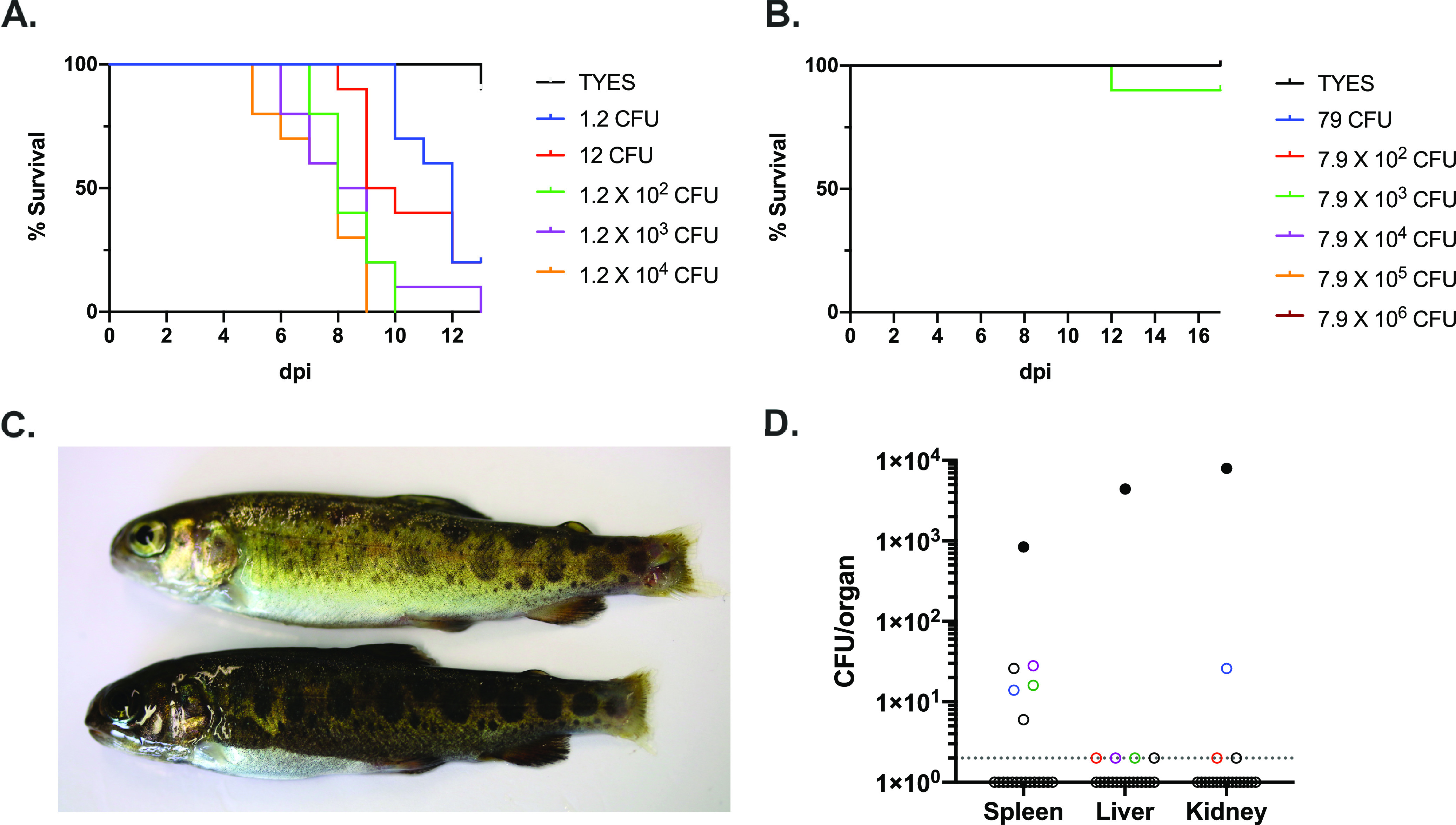
Evaluation of the virulence in rainbow trout. (A and B) Kaplan-Meier survival curves of rainbow trout fingerling (trial 1; Sy line; 6 to 8 g) after intramuscular injection with F. psychrophilum FRGDSA 1882/11 (A) and *F. bernardetii* F-372^T^ (B) bacterial suspensions with doses ranging over 6 orders of magnitude. TYES broth was used as a negative control. (C) Rainbow trout fingerling (trial 1; Sy line) infected with *F. collinsii* TRV642 and attacked by dominant fish display damaged caudal fin and tissue. (D) Bacterial loads in internal organs in rainbow trout fingerling (trial 2; Aut line; 5 g) after intramuscular injection with *F. collinsii* TRV642 using a high infectious dose (7 × 10^6^ CFU). Filled circles, diseased fish euthanized at 13 dpi; open circles, survivor fish sampled at 20 dpi. Colors indicate *F. collinsii*-positive samples retrieved from same fish. The dotted line indicates the bacterial detection threshold.

In an attempt to evaluate the virulence of *F. collinsii* TRV642 and *F. bernardetii* F-372^T^ in rainbow trout, groups of fish from Sy (trial 1) and Aut (trial 2) INRAE reference lines were experimentally infected by intramuscular injection and maintained in flow water at 10°C for assessment of symptoms and mortality. In this experimental infection model, the positive-control strain FRGDSA 1881/11, belonging to the pathogenic species F. psychrophilum, was responsible for high mortality, with a 50% lethal dose (LD_50_) of 4.9 × 10^2^ CFU (trial 1 [Fig. 2A]).

In trial 1, only one fish injected with the median infectious dose of *F. bernardetii* F-372^T^ (7.9 × 10^3^ CFU; Sy line) died at 12 days postinfection (dpi) (Fig. 2B); the fish had no symptoms and internal organs, including the brain, showed no trace of *F. bernardetii*. In trial 2, the experiment was repeated by injecting a group of 10 fish with the highest dose (1.3 × 10^7^ CFU; Aut line), and no mortality occurred. At 20 dpi, internal organs (spleen, liver, kidney and brain) were sampled and streaked on TYES agar (TYESA). No bacterial growth occurred from any organ/fish, indicating that all fish had eliminated injected bacteria. These results suggest that this species is nonpathogenic for rainbow trout under the conditions tested.

In trial 1, one fish injected with the highest dose (1.9 × 10^6^ CFU; Sy line) of *F. collinsii* TRV642 died at 12 dpi and the bacterium was successfully recovered from internal organs of this fish, including spleen, kidney, and liver. We also examined at the end of the experiment internal organs of 13 other fish and observed striking differences linked to the fish status. Indeed, starting from 9 dpi, it was noticed in several tanks that dominant fish of bigger size were attacking smaller fish. This behavior can be observed when fish are maintained in small groups, independently of any challenge. The attacked fish then showed heavily damaged caudal fin and caudal tissue erosion (Fig. 2C), leading to swimming defects. For ethical reasons, the dominant fish was euthanized starting from 14 dpi and the monitoring of mortality was ceased. In some tanks, another fish rapidly became dominant and continued to attack weaker fish. Between 14 and 20 dpi, dominant and attacked fish were euthanized and internal organs were sampled and streaked on TYESA. Out of 8 attacked fish, *F. collinsii* was successfully recovered from internal organs of 7. In sharp contrast, none of the 5 examined dominant fish was positive for *F. collinsii*.

In order to evaluate the possible link between dominance/subordinate status and disease development, an additional experiment was conducted in trial 2. Three groups of 10 fish (Aut line) were infected by injection with a high infectious dose (7 × 10^6^ CFU; 2 groups) or with sterile TYES broth (control group; 1 group). One fish from the infected groups developed injection site necrosis from 7 dpi and was euthanized at 13 dpi to respect humane endpoints. Bacterial examination confirmed the presence of *F. collinsii* in the external lesion, and bacterial loads were >8 × 10^2^ CFU in internal organs (Fig. 2D). No additional mortality occurred. Three dominant fish were observed in the control group, but not in the duplicated infected group. At 20 dpi, all fish of the infected groups were euthanized and internal organs were sampled for bacterial load determination. *F. collinsii* was detected in at least one organ for 9 fish out of 19. Bacterial loads were lower than observed for the diseased fish (Fig. 2D). Taken together, these results indicate that under our experimental conditions, *F. collinsii* TRV642 has very low virulence but is able to survive at low level in internal organs and to produce disease in individuals with compromised conditions such as stress and/or wounds.

## DISCUSSION

Since its thorough 1996 revision on the basis of 16S rDNA gene sequencing (10), the genus *Flavobacterium* has considerably expanded as a result of the growing number of worldwide sampling campaigns (https://lpsn.dsmz.de/search?word=flavobacterium). Most of the *Flavobacterium* species described so far are *a priori* nonpathogenic, environmental isolates, retrieved from a wide diversity of sources (e.g., freshwater and salt water, soil, and rhizosphere). However, a number of newly described species have also been described from fish or fish farm environments, which raised questions on whether some could cause disease (31) like *F. columnare* and F. psychrophilum: *F. columnare* is the causative agent of columnaris disease, which affects a large variety of freshwater fish species generally reared at relatively warm temperatures, such as catfish, tilapia, and ornamental fish (32); F. psychrophilum causes rainbow trout fry syndrome and bacterial cold-water disease, affecting predominantly salmonid reared in cold freshwater. To a lesser extent, bacterial gill disease elicited by *F. branchiophilum* also affects a number of cultured fish species throughout the world (33). However, the last species is considered by some authors an opportunistic pathogen, arising under suboptimal environmental conditions (16). In addition to these species, many *Flavobacterium* species retrieved from diseased fish have been described. A large number of uncharacterized fish-associated *Flavobacterium* spp. were also identified during an 8-year follow-up study of the Laurentian Great Lakes in Michigan (34). However, although these bacteria were indeed retrieved from diseased fish, it was unclear whether the symptoms could actually be attributed to the bacteria that were recovered from the infected fish.

There is a serious lack of studies aiming to demonstrate bacterial virulence using experimental infection models to fulfill Koch’s postulates. It is also unclear which bacteria are true pathogens and which are opportunistic pathogens or even only secondary colonizers, i.e., primarily saprophytic or commensal bacteria able to invade the host only when its defenses are compromised (e.g., by wounds, stress, or disease). This information is crucial for appropriate management measures to mitigate disease emergence and spreading. Here, we report the isolation of *F. collinsii* strain TRV642 from the spleen of a dead rainbow trout raised in an INRAE experimental fish facility. The dead trout did not display any disease symptoms and belonged to a highly F. psychrophilum-susceptible A36 isogenic line that should be considered outstandingly fragile (19).

Whole-genome assembly revealed limited differences in genome size between isolate TRV642 of *F. collinsii* and the type strain, CECT 7796. Strikingly, these genome sizes are far above the mean (4.05 Mbp) and median (3.80 Mbp) genome sizes in the genus *Flavobacterium*. With 5.74 Mbp, strain CECT 7796^T^ possesses the 15th largest genome out of 195 available for the genus (Data Set S1). On the other hand, the two unquestionably fish-pathogenic species F. psychrophilum and *F. columnare* (as well as its newly described sister species *F. covae*, *F. davisii*, and *F. oreochromis*) possess rather compact, about half smaller, genomes. The presumptively opportunistic pathogen *F. branchiophilum* also harbors a relatively small genome of about 3.6 Mbp. The genome of *F. collinsii* is in the same range of size as environmental members of the genus, which suggests an extended ecological niche. Indeed, bacteria living in habitats with diversified nutrient supply tend to have larger genomes and target more complex substrates (35), whereas pathogenicity is generally associated with reduced genome size (36).

The vast majority of the *Flavobacterium* species harboring large genomes are grouped in the phylogenetic tree reconstructed on the alignment of the core genome (Fig. 1), suggesting a common ancestor with a large genome. Since these species were retrieved from a variety of ecological niches (e.g., seawater, freshwater, and soil), it is difficult to formulate a hypothesis for the habitat of this ancestor. Nevertheless, inside or outside this group some subgroups of bacteria isolated from the same type of habitat were identified. In particular, *F. collinsii* tightly clusters with *F. tructae* and several other species (i.e., *F. chilense*, *F. piscis*, *F. bizetiae*, and *F. araucananum*) considered putative fish pathogens because of their isolation from diseased fish. This common source of isolation suggests evolutionarily conserved characteristics. Similarly, a number of species from cold environments, including *F. glaciei*, grouped together elsewhere in the tree.

It is noteworthy that the picture for the habitat of the different species may be blurred by the very limited number of isolates reported for the vast majority of the considered species, which leads us to base our classification essentially on the origin of the type strain. Confining a species to a single habitat may be an oversimplifying hypothesis, especially in the case of the heterotrophic bacteria of the *Flavobacterium* genus. For instance, in 2013, Loch et al. (34) described 32 clusters of isolates belonging to the genus *Flavobacterium* recovered from diseased as well as apparently healthy wild, feral, and farmed fish in Michigan, many of which—most likely representing novel species—displayed significant similarities to environmental species such as F. hercynium, F. pectinovorum, and F. frigidimaris. Furthermore, a 2015 study reported that farmed freshwater fish carried F. suncheonense, F. indicum, F. aquaticum, F. granuli, *F. hercynium*, and *F. terrae*, all previously described as environmental species (37). These studies highlight the extreme diversity of *Flavobacterium* species retrieved from fish and the difficulty-to-identify pathogens.

In line with its large genome, *F. collinsii* TRV642 possesses a broad gene repertoire encompassing 29 PUL likely dedicated to the harvesting of nutrients from the extracellular environment. In addition, the diversity of T9SS cargo proteins, including peptidases and CAZymes, together with other enzymes dedicated to carbohydrate gathering and breakdown, suggests metabolic versatility allowing the bacterium to utilize a large spectrum of carbon sources. Indeed, numerous copies of PUL were identified in environmental species of the family *Flavobacteriaceae*, especially those that are able to utilize a large variety of nutrients sources (e.g., 20 *susCD*-like pairs in Gramella forsetii KT0803, 42 in *F. johnsoniae* UW101, and 71 in Zobellia galactanivorans Dsij), whereas very few pairs were identified in the bona fide fish-pathogenic species (e.g., only 1 *susCD*-like pair in F. psychrophilum JIP02/86, 2 in *F. columnare* ATCC 23463^T^, and 6 in Tenacibaculum maritimum NCIMB 2154^T^). With reference to the Brillat-Savarin’s aphorism “Tell me what you eat, and I will tell you what you are,” one should consider that *F. collinsii* and probably most, if not all, of the aforementioned species occasionally retrieved from fish and harboring large genomes are very likely versatile in their diets. As a result, one might suggest that, as reported for others *Bacteroidota*, these bacteria are associated with niches containing diverse nutrient sources, including eukaryotic organisms, with whom they may have evolved a range of relationships potentially from symbiotic, mutualistic, or commensal to pathogenic interactions. The presence of antibiotic biosynthesis gene clusters in the *F. collinsii* genome was also reported for some mutualistic or commensal bacteria, where they are used to fight other competitors occupying the same niche (38), a phenomenon participating in competitive exclusion.

Using a rainbow trout experimental infection model based on intramuscular injection, which is generally much more efficient than the immersion model since it bypasses natural protection barriers of mucus and skin, neither mortality nor symptoms were observed using the highest dose (1.3 × 10^7^ CFU) of *F. bernardetii* F-372^T^. For *F. collinsii* TRV642, the bacterium produced disease in only 2 fish out of 30 at a high dose (>10^6^ CFU) but was recovered from internal organs (spleen, kidney, and liver) for half of the sampled survivors, including asymptomatic and attacked fish. It is also noteworthy that *F. collinsii* TRV642 was originally isolated from a highly F. psychrophilum-susceptible A36 isogenic rainbow trout line. These results suggest that when *F. collinsii* is able to invade internal tissues, likely through skin lesions, bacterial cells partly resist clearance by the immune system and persist inside the host at a low level. Some fish may be at higher risk of *F. collinsii* infection development due to unknown factors such as host genotype, environmental parameters, coinfections, stresses, and wounds.

In contrast to *F. bernardetii* and *F. collinsii*, experimental infection with F. psychrophilum of the Sy rainbow trout line resulted in high mortality: the median lethal doses (LD_50_s) ranged from ~10^5^ CFU for strains with moderate virulence, such as OSU THCO2-90 (39), to <10^3^ CFU for highly virulent strains as documented for FRGDSA 1882/11 in this study. However, we cannot exclude that different rainbow trout genetic backgrounds, rearing conditions, bacterial growth conditions, or even other *F. bernardetii* and *F. collinsii* isolates could actually produce severe disease.

Many members of the family *Flavobacteriaceae*, and more generally of the phylum *Bacteroidota*, are normal constituents of the host microbiota. However, some of their traits could be considered “dual-use” virulence traits because, while serving important functions for survival in the environment, they can also function as virulence factors (40). Bacteria encompassing these dual-use virulence traits can rapidly proliferate to degrade and metabolize host macromolecules under certain conditions, as recently reported for *Bacteroidota* causing opportunistic diseases in marine eukaryotes (41).

Taken together, our results suggest that some recently described *Flavobacterium* species isolated from fish are likely saprophytic or commensal bacteria that may behave as opportunistic pathogens able to proliferate on decaying tissue to exploit the available nutrients, causing disease under specific circumstances. Indeed, commercially reared fish are subjected to intensive farming practices resulting in welfare issues associated with high stocking density and handling processes that can result in trauma, providing favorable conditions for bacteria to thrive.

## MATERIALS AND METHODS

### Bacterial strains and growth conditions.

Bacteria were grown on tryptone-yeast extract-salt (TYES) broth (0.4% [wt/vol] tryptone, 0.04% [wt/vol] yeast extract, 0.05% [wt/vol] MgSO_4_·7H_2_O, 0.02% [wt/vol] CaCl_2_·2H_2_O, 0.05% [wt/vol] d-glucose [pH 7.2]) or on TYES agar (TYESA) supplemented with 5% fetal bovine serum for 4 days at 18°C. Precultures were prepared using a single colony in TYES broth and grown overnight at 18°C and 200 rpm. Cultures were prepared by dilution of stationary preculture and grown overnight to the early stationary phase (optical density at 600 nm [OD_600_] of 1.0 ± 0.1) for infection challenges. Cultures were 10-fold serially diluted in 1% [wt/vol] peptone water, and CFU were counted *a posteriori* after 48 to 72 h of incubation at 18°C on TYESA. For long-term storage, stationary-phase bacterial cultures were stored at −80°C with 20% (vol/vol) glycerol.

### PCR amplification and 16S rRNA gene sequencing.

PCR amplification was performed using a universal bacterial 16S rRNA gene primer set composed of forward (27F; 5′-AGAGTTTGATCMTGGCTCAG-3′) and reverse (1492R; 5′-TACGGYTACCTGTTACGACTT-3′) primers. The PCR mixture contained final concentrations of 0.3 μM for each primer, 1.25 U of Dream *Taq* DNA polymerase, 1× buffer (Thermo Fisher Scientific), 0.3 mM deoxynucleoside triphosphates (dNTPs), and 1.5 μL of bacterial DNA prepared by heat lysis (at 99°C for 10 min) of stationary-phase culture. The volume was adjusted to 50 μL with double-distilled water (ddH_2_O), and PCR was performed with initial denaturation at 95°C for 5 min followed by 30 cycles of amplification (95°C for 20 s for denaturation, 48°C for 20 s for annealing, and 72°C for 2 min for extension) and finished by extension at 72°C for 10 min. PCR amplicons were sequenced by Sanger sequencing. A specific *F. collinsii* PCR detection, targeting FLACOL7796_04448 CDS (UniProt no. A0A6J4GVB1), was developed using primers TRO1016 (5′-TTTCATGACGCATTGCTGCC-3′) and TRO1017 (5′-AAAATTTTCCCCGGCACACG-3′). The PCR mixture was prepared as for the 16S rRNA gene, and PCR was performed with initial denaturation at 95°C for 5 min, 30 cycles of amplification (95°C for 30 s for denaturation, 52°C for 30 s for annealing, and 72°C for 1 min for extension), and final extension at 72°C for 10 min. A positive *F. collinsii* PCR amplification provided a 479-bp band.

### Rainbow trout experimental infections.

Two trials were conducted using rainbow trout reference INRAE lines (42): Sy line (trial 1) and Aut line (trial 2). Fish were reared at 10°C in a recirculating aquaculture system (RAS) in 30-L tanks with dechlorinated tap water. Two days before infection, fish were transferred to the biosafety level 2 (BSL2) zone in 15-L tanks with flow water (1 renewal per hour). Experiments were carried out for *F. bernardetii* F-372^T^, *F. collinsii* TRV642, and F. psychrophilum FRGDSA 1882/11 using rainbow trout fingerlings (weight, 5 to 8 g).

### (i) Trial 1.

Experimental infections with *F. bernardetii* F-372^T^ and *F. collinsii* TRV642 were performed using 7 groups of 10 fish (Sy line) for each bacterial species. In each challenge, one group of 10 fish was injected with sterile TYES broth as a negative control. Six groups were challenged at different doses, as follows: 6 suspensions were prepared by 10-fold serial dilutions (10^−1^ to 10^−6^) of a bacterial culture, and then 50 μL of each suspension was administered per fish by intramuscular injection at a point midway between the insertion of the dorsal fin and the lateral line following anesthesia (50 mg/L; MS222; Sigma-Aldrich). For F. psychrophilum, only 5 groups corresponding to 10^−3^ to 10^−7^ dilutions were used and the median lethal dose was estimated using the moving average and interpolation method described by Thompson (43), with 3 dose groups used to calculate each moving average.

### (ii) Trial 2.

Experimental infections with *F. bernardetii* and *F. collinsii* were repeated independently using the rainbow trout Aut line and the highest dose using 4 groups of 10 fish injected with sterile TYES broth (1 replicate; negative control), *F. collinsii* TRV642 at 10^−1^ dilution (2 replicates), or *F. bernardetii* F-372^T^ at 10^−1^ dilution (1 replicate). All fish were maintained using flow water at 10°C. Mortality was recorded twice a day, and fish were monitored for clinical signs and behavioral changes using a scoring grid throughout the challenge. For each tank, 5-min periods of direct observations performed before feeding were used to detect the presence of dominant and subordinate fish and to record individuals exhibiting external signs, including skin lesions and fin erosion. Dominance was recorded based on aggressive behavior, such as unidirectional sharp movement directed toward another fish, associated with biting.

Euthanasia was carried out by bathing fish in tricaine at concentration of 300 mg/L (MS222; Sigma-Aldrich). Dead or euthanized fish were examined for the presence of bacteria in organs including the spleen, kidney, and liver. Organs were homogenized in peptone water containing silica spheres using a FastPrep instrument (MP Biomedicals) at 6 m/s for 20 s. Tissue homogenates were streaked on TYESA and kept for 3 to 5 days at 18°C before examination for bacterial growth. The identification of *F. collinsii* colonies was performed by specific PCR detection.

### Ethics statement.

Animal experiments and sampling were performed in accordance with European Directive 2010/2063/UE. All animal work was approved by the Direction of the Veterinary Services of Versailles, France (authorization number C78-720), and by the ethics committee of the INRAE Center in Jouy-en-Josas (COMETHEA no. 45), France (authorization no. 2015100215242446).

### Sequencing and annotation of the *F. collinsii* genome.

Genomic DNA (gDNA) was extracted from stationary-phase broth culture using a genomic DNA-Tip 100/G system and buffer set (Qiagen) following the manufacturer’s instruction. For short-read sequencing, the library was constructed using a TruSeq genomic kit (Illumina) and paired-end sequenced on a NextSeq instrument using a NextSeq 500/550 mid-output kit v2 (Illumina). In parallel, gDNA was sequenced on GridION (Oxford Nanopore) using a FLO-MIN106 flow cell for long-read sequencing. Hybrid genome assembly was performed using Unicycler v0.4.8, available on the PATRIC platform, with default parameters (44). Genome annotation and comparison were performed using the MicroScope platform (45). InterProScan was used to identify SusCD pairs using IPR023996 and IPR023997 for SusC and IPR012944, IPR024302, IPR041662, and IPR033985 for SusD with colocalization criteria. Proteins secreted by the T9SS harboring conserved C-terminal domains (CTDs) were identified using IPR026444 and IPR026341 for type A and B CTDs (46), respectively. Carbohydrate-active enzymes (CAZymes) were identified using dbCAN, which combines three tools; hits found by only one tool were removed to improve accuracy (47). Peptidases were identified using the MEROPS database (https://www.ebi.ac.uk/merops/) (48).

### Phylogenetic analysis.

Average nucleotide identities (ANIs) were computed using OrthoANIu and the calculator available at http://www.ezbiocloud.net/tools/ani (49).

Genome records were retrieved from the RefSeq database (https://www.ncbi.nlm.nih.gov/datasets/genomes/?taxon=237&utm_source=data-hub [accessed April 2022]) (50).

Proteomes were compared pairwise with BLASTP (v2.12, low-complexity filter disactivated, E value cutoff of 1e−5, otherwise default settings) (51) and the results served for a single linkage clustering of the genes based on a criterion of 45% amino acid sequence identity over 70% of the gene length. Conserved single-copy genes were identified as clusters with a single representative in each genome and their sequences were subjected to multiple-sequence alignment using Muscle (v3.8.31, default setting) (52).

Neighbor-joining phylogenetic tree reconstruction was conducted using FastTree (v2.1.10, default settings) (53) on the concatenated multiple-sequence alignments, after alignment gap removal. Statistical support of the tree topology was assessed by comparison with trees reconstructed on 400 bootstrap replicates of the original alignment.

### Data availability.

The annotated TRV642 genome sequence has been deposited at ENA under accession number OX336425.1.
