# Supplementary material for: Investigation of the Genus Flavobacterium as a Reservoir for Fish-Pathogenic Bacterial Species: the Case of Flavobacterium collinsii
Source: Appl Environ Microbiol. 2023 Mar 28;89(4):e02162-22. doi: 10.1128/aem.02162-22 (PMC10132118; doi:10.1128/aem.02162-22)
Supplement: Supplemental file 1 — Fig. S1. Download aem.02162-22-s0001.pdf, PDF file, 0.1 MB [file aem.02162-22-s0001.pdf]

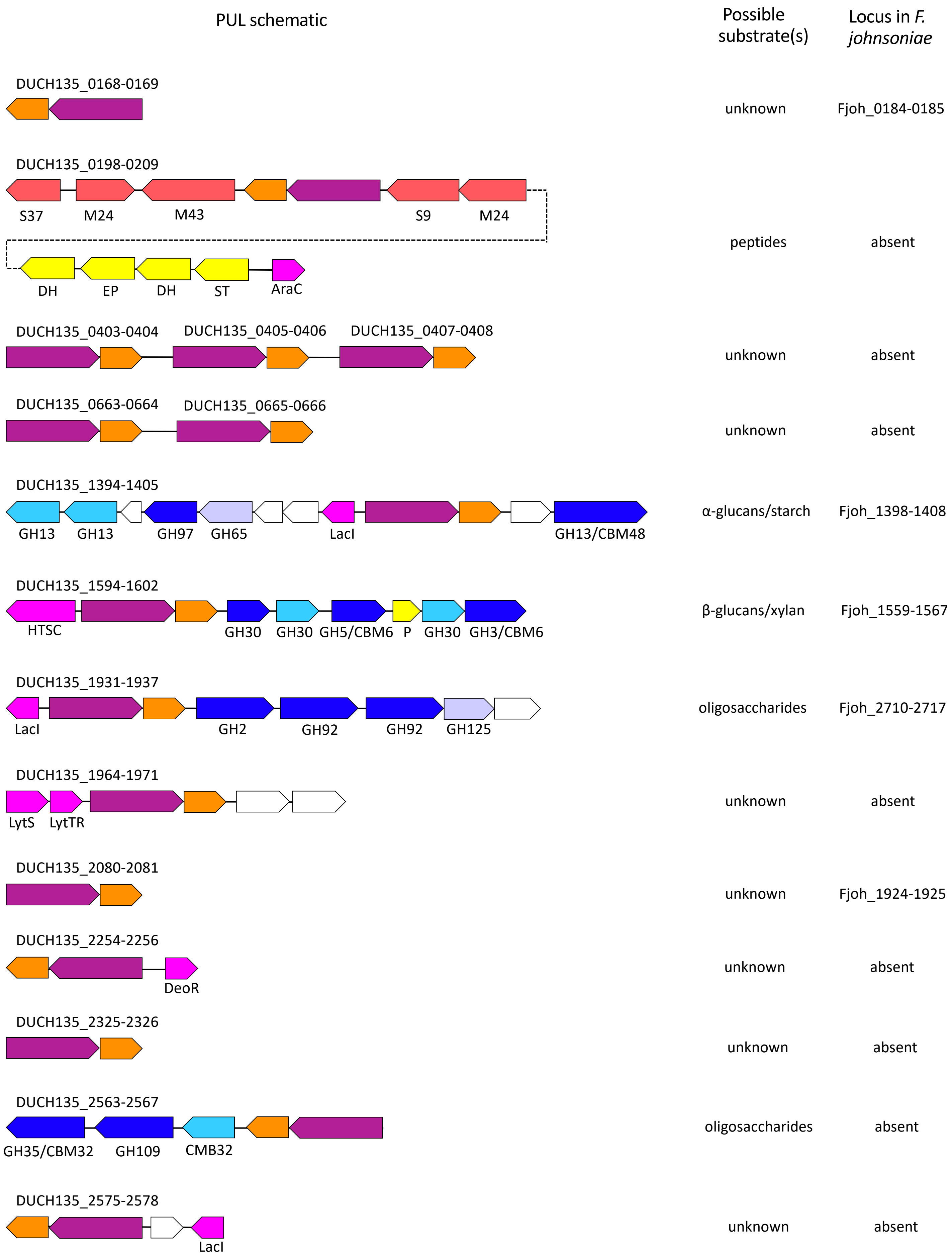

PUL schematic
Possible substrate(s)
Locus in *F. johnsoniae*
DUCH135_0168-0169
unknown
Fjoh_0184-0185
DUCH135_0198-0209
S37
M24
M43
S9
M24
DH
EP
DH
ST
AraC
peptides
absent
DUCH135_0403-0404
DUCH135_0405-0406
DUCH135_0407-0408
unknown
absent
DUCH135_0663-0664
DUCH135_0665-0666
unknown
absent
DUCH135_1394-1405
GH13
GH13
GH97
GH65
LacI
GH13/CBM48
α-glucans/starch
Fjoh_1398-1408
DUCH135_1594-1602
HTSC
GH30
GH30
GH5/CBM6
P
GH30
GH3/CBM6
β-glucans/xylan
Fjoh_1559-1567
DUCH135_1931-1937
LacI
GH2
GH92
GH92
GH125
oligosaccharides
Fjoh_2710-2717
DUCH135_1964-1971
LytS
LytTR
unknown
absent
DUCH135_2080-2081
unknown
Fjoh_1924-1925
DUCH135_2254-2256
DeoR
unknown
absent
DUCH135_2325-2326
unknown
absent
DUCH135_2563-2567
GH35/CBM32
GH109
CMB32
oligosaccharides
absent
DUCH135_2575-2578
LacI
unknown
absent

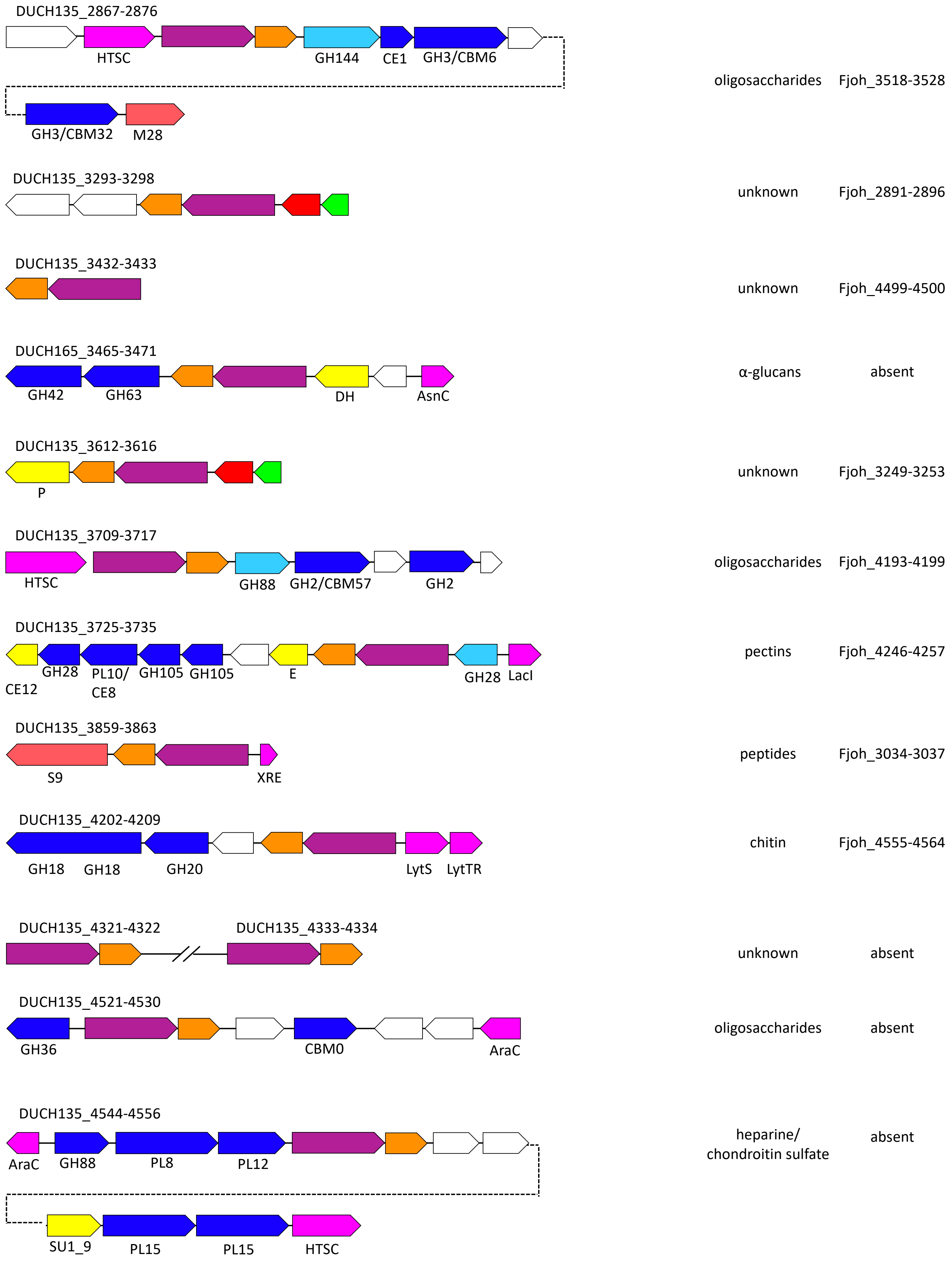

DUCH135_2867-2876
HTSC
GH144
CE1
GH3/CBM6
GH3/CBM32
M28
oligosaccharides
Fjoh_3518-3528
DUCH135_3293-3298
unknown
Fjoh_2891-2896
DUCH135_3432-3433
unknown
Fjoh_4499-4500
DUCH165_3465-3471
GH42
GH63
DH
AsnC
α-glucans
absent
DUCH135_3612-3616
P
unknown
Fjoh_3249-3253
DUCH135_3709-3717
HTSC
GH88
GH2/CBM57
GH2
oligosaccharides
Fjoh_4193-4199
DUCH135_3725-3735
GH28
PL10/
CE8
GH105
GH105
E
GH28
LacI
CE12
pectins
Fjoh_4246-4257
DUCH135_3859-3863
S9
XRE
peptides
Fjoh_3034-3037
DUCH135_4202-4209
GH18
GH18
GH20
LytS
LytTR
chitin
Fjoh_4555-4564
DUCH135_4321-4322
DUCH135_4333-4334
unknown
absent
DUCH135_4521-4530
GH36
CBM0
AraC
oligosaccharides
absent
DUCH135_4544-4556
AraC
GH88
PL8
PL12
SU1_9
PL15
PL15
HTSC
heparine/
chondroitin sulfate
absent

Key to labels:

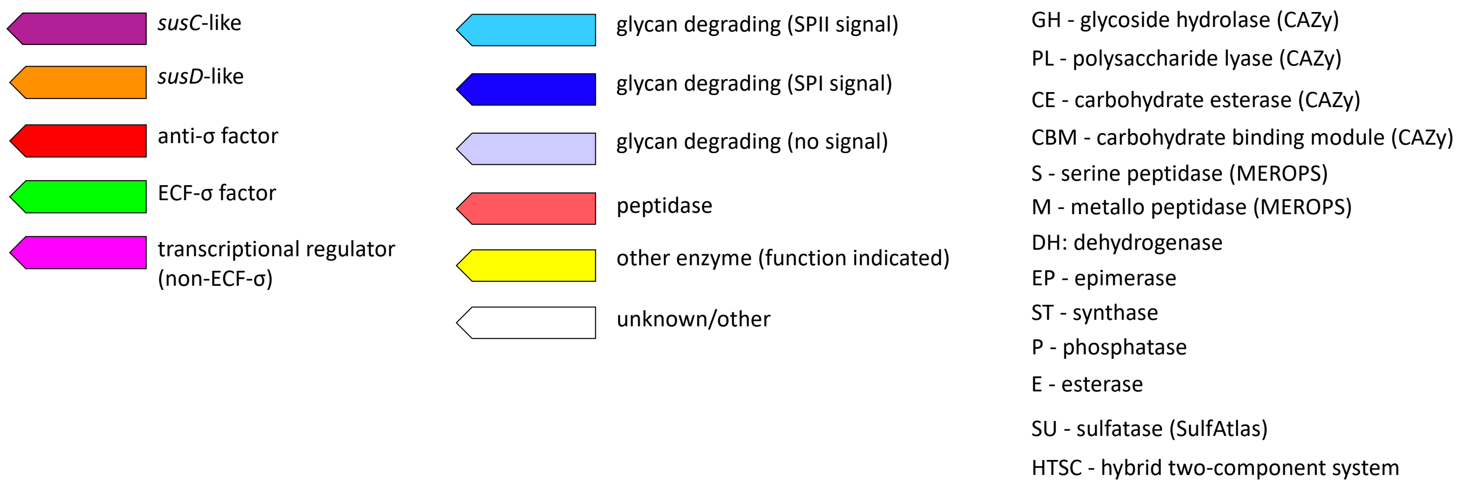

Fig S1. Polysaccharide utilization loci (PULs) and other macromolecule utilization loci of *F. collinsii* TRV642 containing *susCD*-like gene pairs.

Putative functions are labeled as indicated in the key. Gene product functions are labeled according to their respective glycoside hydrolase (GH), polysaccharide lyase (PL), carbohydrate esterase (CE), carbohydrate binding module (CBM) family from the CAZy database or peptidase (P) from the MEROPS database. Possible substrates are indicated based on the specificities of each system. If present, corresponding loci in the *F. johnsoniae* UW101 genome are indicated.
